# Integration of origami and deployable concept in volumetric modular units

**DOI:** 10.1038/s41598-022-18951-w

**Published:** 2022-11-10

**Authors:** Valentina Beatini, Perampalam Gatheeshgar, Heshachanaa Rajanayagam, Keerthan Poologanathan, Thadshajini Suntharalingam, Dilini Perera, Elilarasi Kanthasamy, Brabha Nagaratnam

**Affiliations:** 1 Department of Civil and Architectural Engineering, Aarhus University, Aarhus, Denmark; 2 School of Computing, Engineering and Digital Technologies, Teesside University, Middlesbrough, UK; 3 Department of Mechanical and Construction Engineering, Northumbria University, Newcastle Upon Tyne, UK

**Keywords:** Civil engineering, Mechanical engineering

## Abstract

Modular building systems (MBS) and Origami are two emerging methods used in current construction practice. Origami is directly associated with the principles of the ancient Japanese art of paper folding, characterised by high morphological possibilities and ultimately creates foldable structures with tuneable mechanical properties. However, there is a lack of knowledge on the structural behaviour of origami for architectural engineering applications. MBS is a volumetric prefabricated construction technique enhancing productivity in construction. In this paper, a modular unit is designed which employs origami techniques. The roof and floor panels of the modular units formed with steel joists were substituted with origami sandwich panels, while corner posts were substituted with origami columns. The origami-like foldable system demonstrated superior efficiency in constructability, being highly compact during transportation and requiring few operations for the in-situ installation. The structural performances of the developed and foldable modular units were assessed through finite element analysis. It was found that, without increasing the self-weight of the system, the design of origami-like modular units can be tuned for high structural performances and various structural sizes, which can impact the usability of space and the aesthetics of architecture. While this is a preliminary study and physical testing is needed, the positive results open the possibility of exploring highly deployable modular structures of novel shapes that can be employed during post-disaster and emergencies (Covid-19).

## Introduction

**Figure 1:**
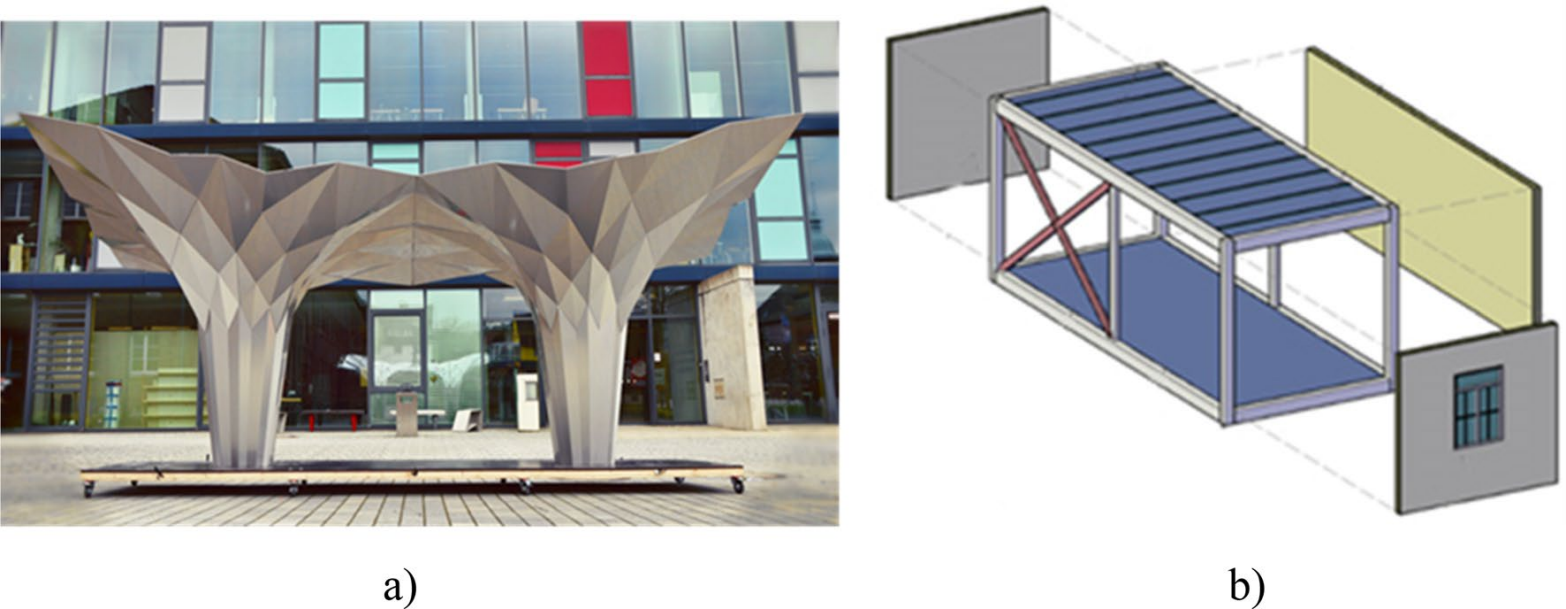
Example of (**a**) origami-inspired steel pavilion building prototype^9^ and (**b**) typical modular building.

Modular building is an emerging construction method, where the construction work of a self-standing structural room or part of a room is performed in the external environment (factories) and leaves the assembly of more modules to be completed onsite^1^. Compared to the traditional onsite construction methods, modular building systems (MBS) can offer efficient material consumption^2^, up to 70% less construction time^3^ and 20% reduced cost with less workforce^4,5^, improved construction quality with desired structural stability^6^, less construction wastage (up to 90%)^7^, reduced energy consumption and reduced noise pollution (30–50%)^2^. From an architectural viewpoint, the most advantageous ones are corner-supported modular units (Fig. 1b). Here, a pre-engineered structural frame is constructed that allows the sides of the room to be open. Though modular construction has experienced steady growth over the years, an accelerated expansion was observed during the covid-19 pandemic. This is due to the demand for reliable structures within a short period of time required for the quick erection of healthcare infrastructure^8^. Despite these advantages, modular units suffer transportation difficulties^1^, restricting their weight, span, height, and width. To maximise the constructed volume, the unit is standardised and parallelepiped, consequently reducing the architectural design freedom and the societal appeal of the technology.

Deployable structures are structures that change shape and size. Among the methods to obtain them, origami and kirigami derive their principles from the ancient Japanese art of paper folding and paper folding and cutting, respectively^10^. The application of the origami technique is gaining attention in buildings as seen in Fig. 1a. The combined use of origami and modular would offer more innovation to the construction.

The authors believe that the origami technique can be a solution to overcome the transportation limitations inherent to the modular building field, thanks to the modularity and folding/unfolding capabilities that it offers. This study is a first attempt to substitute the structure of the corner-supported modular building with rigidly foldable origami ensuring the path towards implementing more diverse morphology of origami designs like those envisioned by various research^11–13^. In the following section, the origami technique to validate the hypothesis is presented, followed by the analysis of the results, where the viability of a novel origami-like modular unit is discussed against a corner-supported MBS. Subsequently, the model development section is discussed. In conclusions the limitations of the research including future work is mentioned, which could improve the system's desirability.

## The origami technique

### Origami for MBS applications

**Figure 2:**
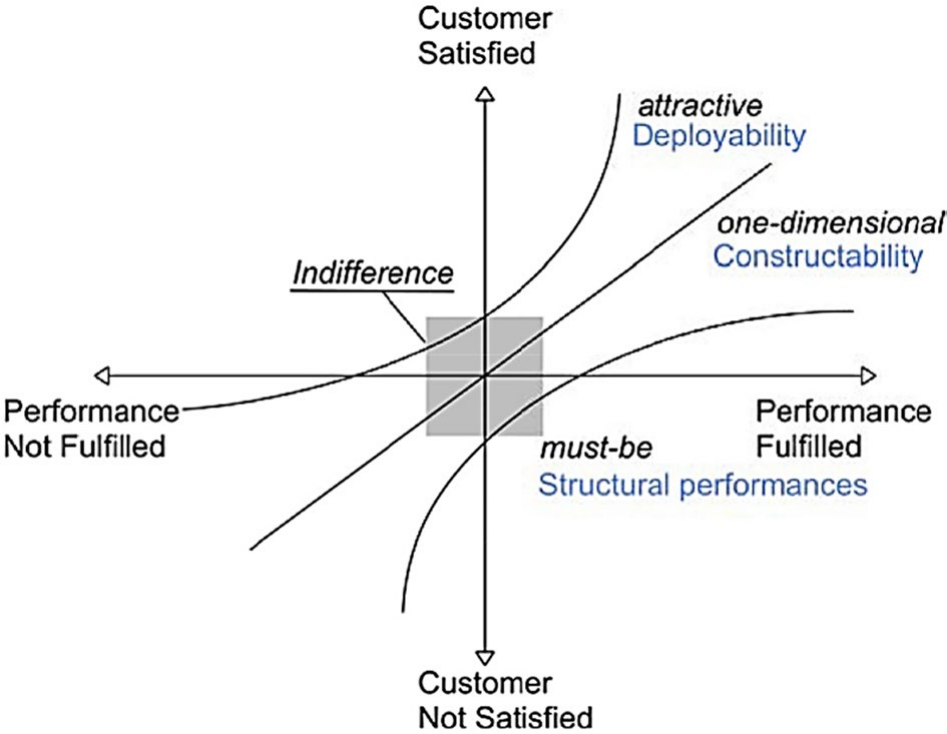
The Kano model^14^.

The Kano model (Fig. 2) for product development has been used to identify the origami principles and technical aspects relevant to the envisioned application. Accordingly, the design requirements are divided into an attractive, one-dimensional and must-be requirements^14^. The attractiveness of origami-like modular units lies in the modular, deployable subcomponents, which facilitate transportability and allow for design variation. This often refers to constructability of the modular unit and identified as a one-dimensional requirement, as it satisfies the market proportionately to its performance. In addition, the origami system must also meet the structural requirements of the modular building systems.

#### Deployability

When it comes to engineering applications, rigid origami^15^ are typically employed. This is a system which can be folded and unfolded without cutting or deforming the material except where the folds are made. The pavilion depicted in Fig. 1a is a rigid origami made of steel. Its modular design illustrates the morphological possibilities of origami, which are due to slight adjustments within the planar angles forming the plates. It is possible to obtain origami approximating target curves and surfaces through reverse engineering processes^16^. Within the mechanical design field, rigid origami are examples of spherical linkages: the fold lines are hinge joints, and the planar angles within the plates represent the length of the links^17^. The rotatability of the origami depends on the latter^18^. A minimum origami mechanism has four plates sharing a vertex. As the number of plates sharing a vertex increases, so does the degree of freedom (DOF). This is the number of input variables (usually the rotation of the hinges) that must be independently controlled to bring the mechanism into the desired working configuration. The ease of control of the deployment process depends on this mechanism. The depicted pavilion has many DOFs, which caused its originally foldable ribs to be fully fastened to each other during manufacturing to facilitate erection. For the case at hand, it is crucial to note that adequately designed origami tessellations whose interior vertexes have valence four create mechanisms with 1-DOF^19^.

#### Constructability

In rigid origami, the plate material is irrelevant to the kinematics and can be designed to achieve the desired performance, such as in the acoustic chamber proposed^20^. However, geometrical incompatibilities may emerge during bending due to the material's thickness^21^. In this regard, a distinction shall be made between ‘strict’ origami, derivable by solely folding a sheet of material^15^, and origami-like structures, where more plates are joined a posteriori. In the former case, compliant joints can be employed, as inside each ¼ rib of the pavilion depicted in Fig. 1. This allows to lower the number of manufacturing independent processes, and the structure can be more lightweight. The whole pavilion is an ’origami-like’ structure obtained by joining the eight original sheets of posteriori. The fastening elements connecting the pavilion’s eight ribs are located on outward-pointing flaps. Alternatively, rubber joints have also been proposed^22^. Both these methods have drawbacks, as they can increase the weight and volume of the structure or have limited structural performances respectively.

#### Structural performances

**Figure 3:**
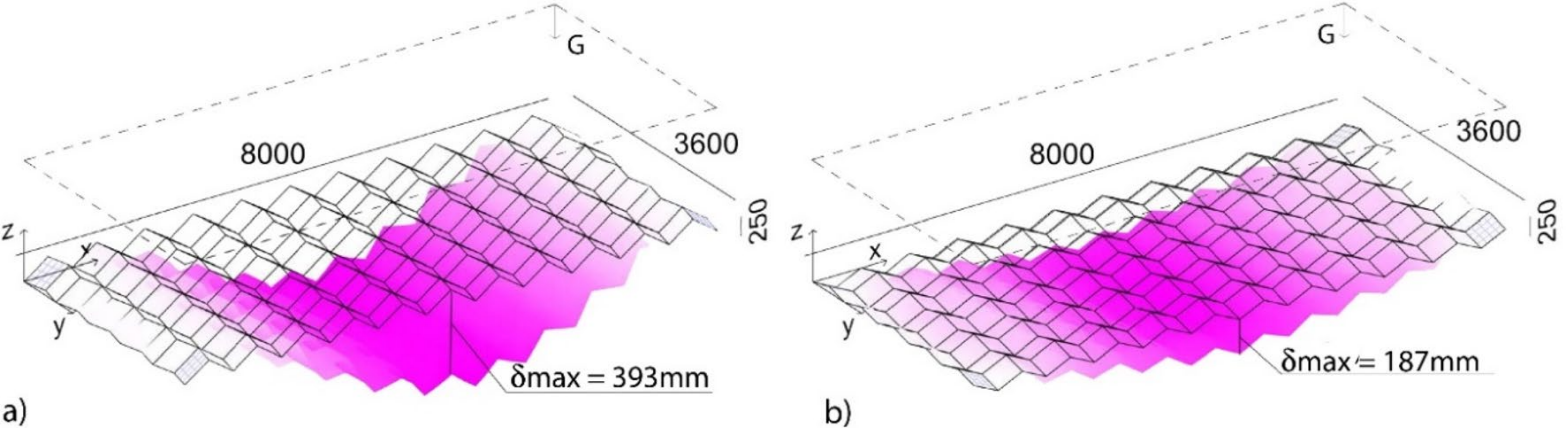
Deflection of (**a**) a Miura Ori tiling (a strict origami) and (**b**) an egg-box tiling (an origami-like structure) under self-weight.

The few architectural applications of origami are temporary pavilions, and there is a lack of knowledge on the suitability of origami for structural applications at the architectural scale. As a preliminary understanding, we examine 1-DOF assemblies containing a strict origami (a Miura pattern) and an origami-like tiling (an egg-box pattern) under gravity loads, which are MBS's governing loading conditions. These patterns were organized in a grid of 10 × 22 plates, altogether having dimensions of 8000 mm (L, length) × 3600 mm (W, width) × 25 mm (H, height), comparable to those of MBS floors. The plates were assigned a thickness, *t* of 5 mm and assumed to be rigidly connected to each other. The corner plates were fixed, and the resulting structures were subjected to self-weight. Here and in the following section, the considered samples were modelled in a parametric environment within the Rhino@ software^23^ and analysed with the Finite Element method implemented through the Karamba plugin^24^ considering the effects of second order. For all the analyses, the material was assumed to be steel having a modulus of elasticity *E* of 210 GPa, modulus of rigidity *G* of 80.76 GPa, density γ of 78.5 kN/m^3^, yield strength *fy* of 360 MPa. Every origami plate was meshed into 32 triangles using shell elements. The results are evaluated based on structural efficiency. These one-layer structures deflected beyond acceptable limits as shown in Fig. 3. The origami-like structure, with a mass, *m* of 1270 kg, had a maximum displacement, *d*_max_ of 187 mm, which equals to L/43. The Miura pattern, with a mass *m* of 1383 kg, reported a maximum displacement, *d*_max_ of 393 mm, equal to L/20 and almost doubled the one of the egg-box pattern. While the analysis is not exhaustive of the many possible designs, the results highlight that the strict origami surface can fold back to a flat surface without breakage or extensional deformation, so it lacks geometrical stiffness. Besides, despite the structures being lightweight, it would be not easy to fold onsite steel sheets with a thickness *t* = 5 mm.

**Figure 4:**
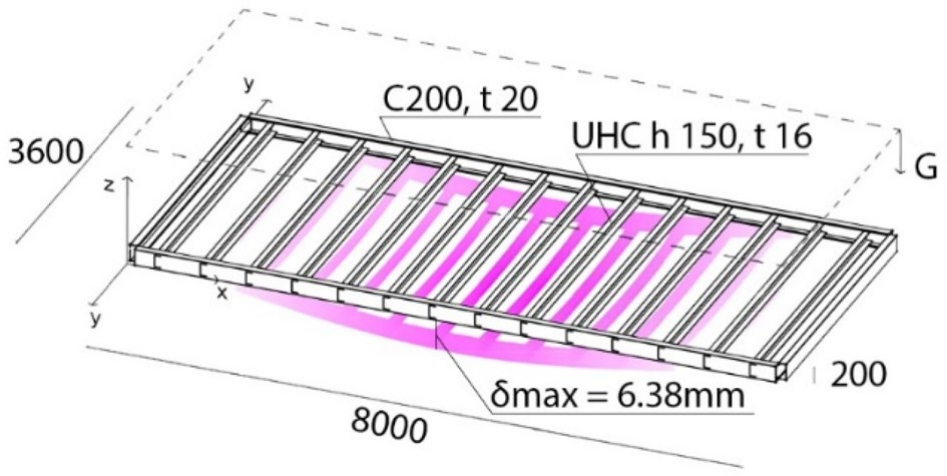
Deflection of a typical MBS roof under self-weight.

To better visualise the problem, a typical steel roof of an MBS was modelled with shell elements and subjected to self-weight, Fig. 4. The floor has dimensions 8000 mm (L) × 3600 mm (W) × 20 mm (H). The floor panel joists are lipped channels 150 mm × 75 mm × 17 mm × 1.6 mm; the bearers are unlipped channels with a depth of 200 mm and thickness of 2.0 mm. The free edges of the bearers are modelled as rigid supports. The structure has a mass *m* of 3330 kg and reported a maximum displacement *d*_max_ of 6 mm, equal to L/1245. This analysis demonstrates the need to improve the geometrical stiffness of origami and that the increased stiffness of the origami-like structure versus strict origami is not substantial to the application’s success.

## Results and discussion

### Competitor benchmarking

**Figure 5:**
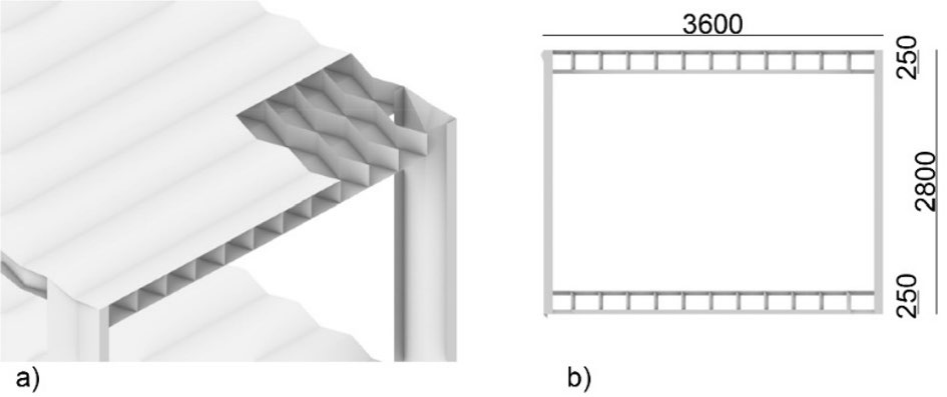
The proposed origami-like modular unit. (**a**) Axonometry. (**b**) Side elevation.

Figure 5 illustrates the proposed origami-like modular unit. It is a 1-DOF mechanism employing rigid origami and is made of steel. The floor and roof are corrugated sandwich structures composed of strict origami. Given the demonstrative aim of the paper, the classic Miura pattern was selected. By composing it in a sandwich panel, the geometrical stiffness of the structure is increased without sacrificing the constructional advantages. *C*-like foldable columns connect the floor and roof. The feasibility of the solution is tested through a competitor benchmark^25^.

### Deployability

Considering a standard modular unit, it was made deployable by inserting hinges. These are 1-DOF joints, which minimise erection errors, and are less prone to malfunction due to dust. The joints were placed to ensure that the modular unit fits the dimensions of the roadway during transportation and in such a way that the longitudinal structural span of the modular unit may be widened. The result is a standard modular unit with detached deployable floor and roof sections. The mechanism may allow the MBS to be longer but not change its shape.

The deployability of an MBS can be highly improved by employing origami techniques. As mentioned in “The origami technique” section, properly designed origami tessellations with valence-four vertexes ensure that the mechanism has 1-DOF. As detailed above, the floor and roof are designed accordingly. As discussed in the “Conclusions”, it would be further possible to value the morphological possibilities of origami to broaden the design solution space.

### Constructability

**Figure 6:**
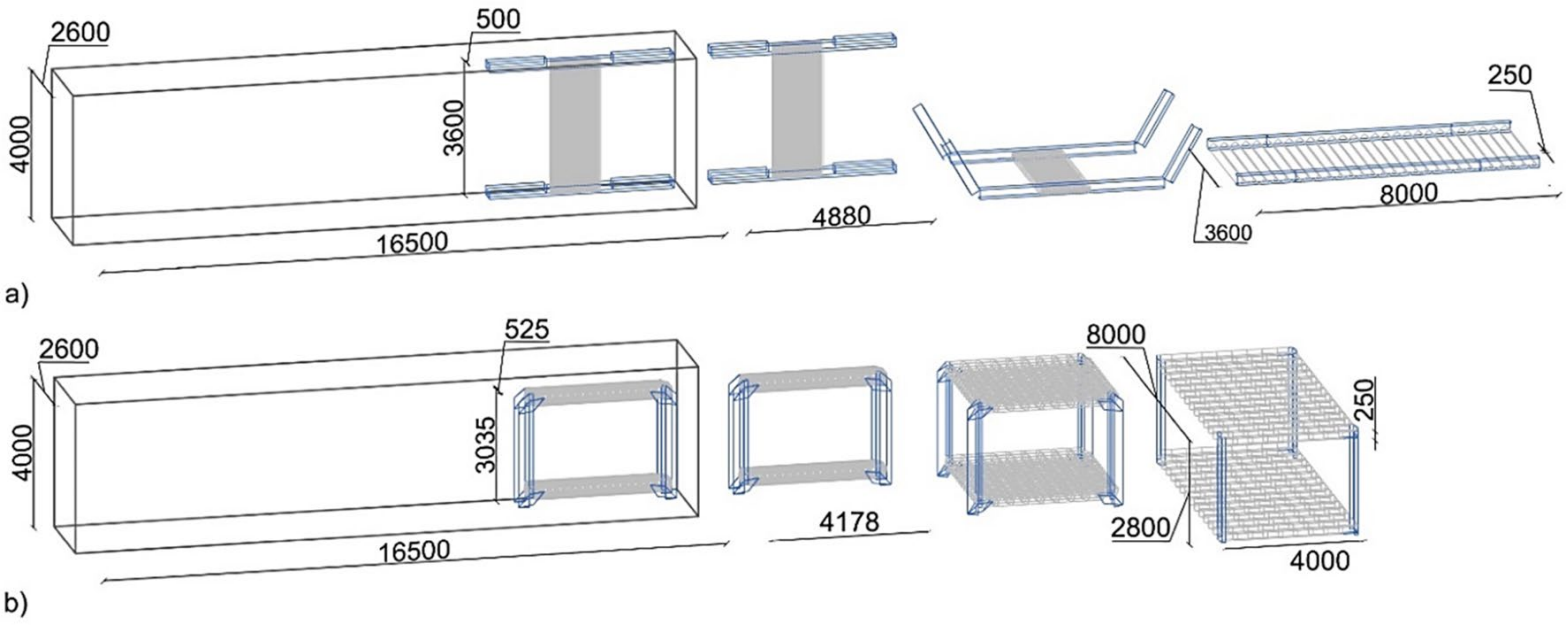
Considered deployable modular sections and structural dimensions. (**a**) Deployable floor and roof. (**b**) Modular origami-like unit.

This analysis aims to understand the possible incremental advantages of the origami-like unit. The construction process of the proposed systems varies substantially. Whereas the kinetic standard modular design, Fig. 6a, is composed of 6 prefabricated deployable components (roof, floor, and four columns) and assembled on-site, the modular origami-like unit, Fig. 6b, is wholly prefabricated off-site and folded on-site.

Compared to a standard MBS, both the deployable and the foldable origami-like systems demonstrate superior transportation economy and increased sustainability. Assuming as a reference volume of a trailer the maximum allowable volume for road transportation in Europe, which is 16.5 m (L) × 2.6 m (W) × 4 m (H), Fig. 6a,b show the space occupied by one deployable roof/floor and one origami-like unit, respectively. The former has dimensions 4.88 m (L) × 0.50 m (W) × 3.6 m (H) in its compact state, whereas the same dimensions in the origami-like unit read 4.18 m × 0.53 m × 3 m. A single trailer could transport about 12 origami-like modular units or 12 deployable roofs/floors.

### Structural performances

**Figure 7:**
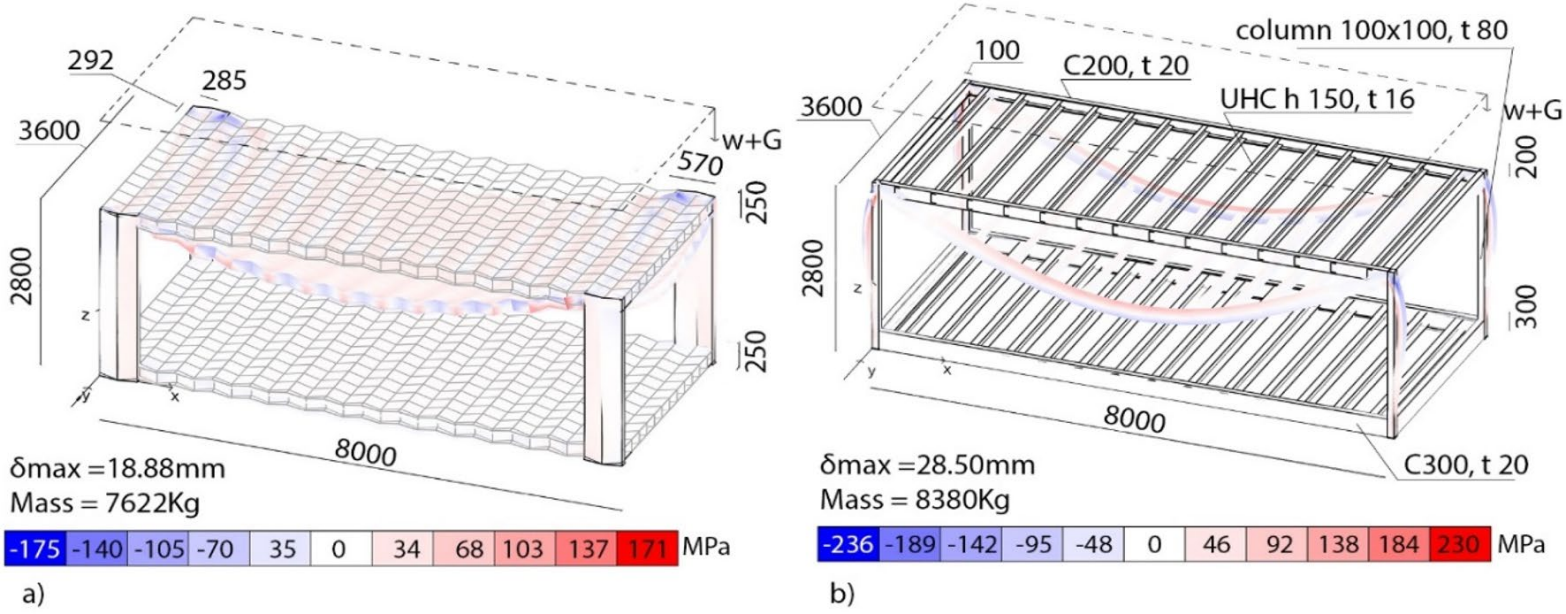
Structural behaviour of an origami-like unit (**a**) and a standard MBS (**b**) under self-weight and uniform gravity load equal to 5 kN/m^2^.

Figure 7a illustrates one of the possible solutions that can be obtained from varying the geometrical parameters of the solution identified. The sandwich floor/roof of the origami-like modular system employs plates having a constant thickness, *t* of 2.0 mm. By construction, the core layer has a thickness of *t*; the facings have a thickness of 2*t*, which increases their bending rigidity. The *C*-like columns have a thickness, *t* of 8.0 mm, a flange length of 100 mm, and a channel length of 571 mm, which matches the span of the connected modular floor. This last length can be varied according to needs. Its structural performances are compared to the ones of a standard modular building. For the standard system, Fig. 7b, the floor panel joists are lipped channels 231 mm × 75 mm × 17 mm × 1.6 mm, while the roof panel joists are lipped channels 150 mm × 75 mm × 17 mm × 1.6 mm. The bearers are unlipped channels with depths respectively 200 mm and 250 mm and 2.0 mm thickness. The columns have a square cross-section of 100 mm × 100 mm and thickness *t* = 8 mm. The dimensions of the module are L × W × H = 8000 mm × 3600 mm × 2800 mm. Both cases are subjected to a uniformly distributed load w = 5 kN/m^2^ applied on the top floor (roof) and self-weight. As detailed earlier, steel has been used in the analyses. The study highlights the potential benefits of the origami-like unit in terms of structural efficiency. In the origami-like unit, the maximum deflection is 18.88 mm, the peak Von Mises stress is − 1758 MPa, and the mass is 7622 kg. The same values in the standard MBS read 28.50 mm, − 236 MPa and 8380 kg, respectively.

## Model development

### Problem formulation

**Figure 8:**
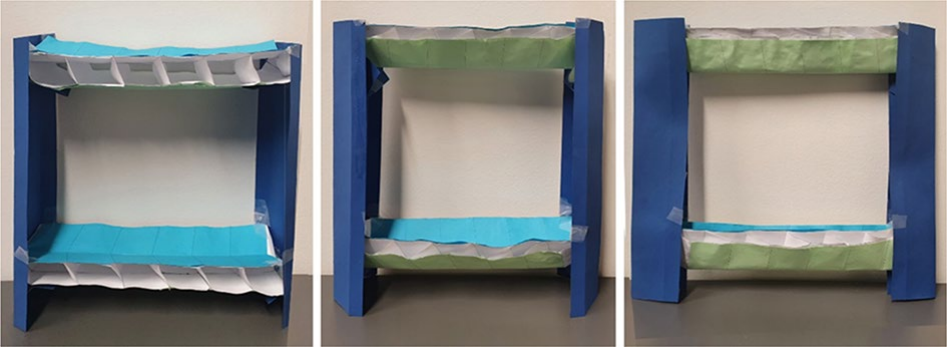
Concept model of the proposed system at different folding states.

Leveraging on the observations made, the design process aimed at constructing a highly foldable, corner-supported origami modular unit employing strict origami and able to withstand the structural loads typical of MBS structures. Figure 8 illustrates two columns and short floors and exemplifies the concept design of the proposed origami-like modular unit. The transversal span of the cells composing the floor decreases while folding, and as such, the system can fold to a very compact volume regardless of its longitudinal dimensions. The columns stick to the ground throughout the folding/unfolding process.

As discussed in “The origami technique” section, one-layer origami structural surfaces lack geometrical stiffness. Consequently, the proposed roof and floor are sandwich structures. The technical constraints set by the sandwich panel were preliminarily envisioned to ensure the feasibility of the subsequent design decisions.

**Figure 9:**
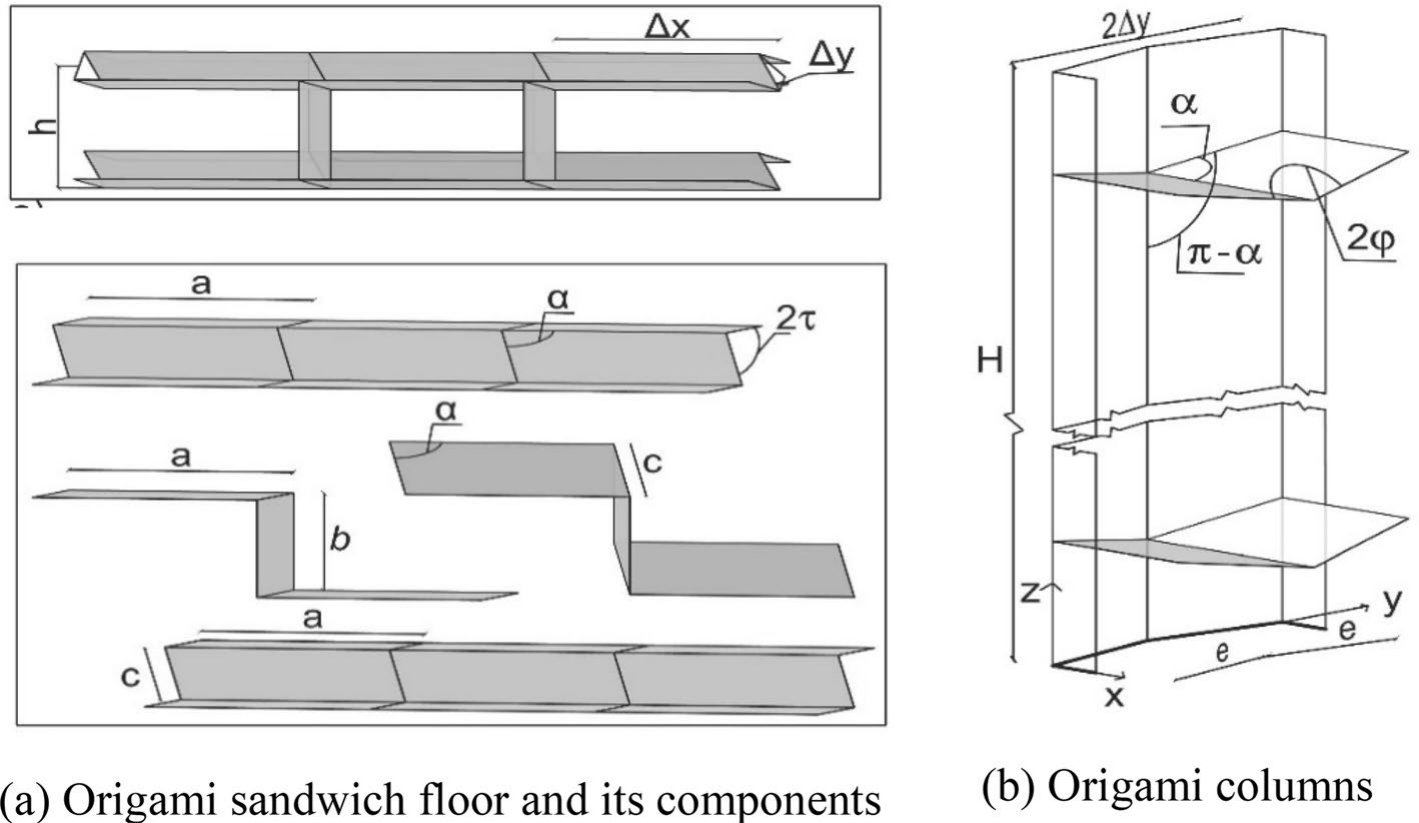
Proposed origami sandwich floor/roof and origami columns for the origami-like modular unit. (**a**) Origami sandwich floor and its components. (**b**) Origami columns.

**Figure 10:**
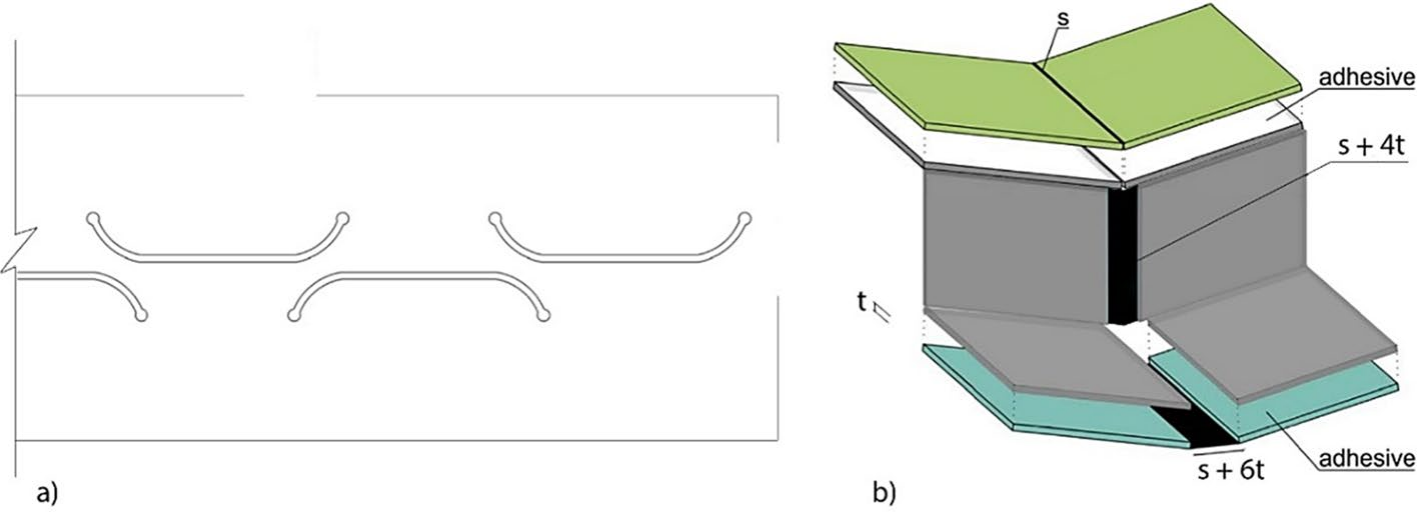
(**a**) A steel compliant joint^30^. (**b**) The space allocated to the compliant joints and the position of the adhesives within the origami sandwich floor/roof.

By adequately combining foldable origami obtained from three sheets of material, Gattas and You^26^ proposed a stiff origami-like kinetic sandwich structure with a triangular core. However, the constraints set by that mechanism impose linear contacts between the sheets composing the core and those composing the faces of the panel, which make its manufacturing quite complex. De Waal et al.^27^ presented a foldable honeycomb structure where the sheet composing the core creates a surface contact with the sheets composing the face layers. This type of contact can be implemented using adhesives: it is easier to be manufactured and is prone to fewer manufacturing errors^28^. The sandwich panel proposed here, Fig. 9a, is composed of three sheets with face contact. They are organised so that, at the target motion configuration *C*, the cells of the hollow core have a rectangular profile. The upper and lower sheets have a simple concertina pattern, while the mid one has a Miura pattern. Accordingly, compliant joints can be used within each layer, and adhesives can be used to connect the three layers, Fig. 10b. A compliant joint made of steel is depicted in Fig. 10a. Its exact shape will be evaluated in a successive study. To accommodate the thickness of the material throughout the folding process, Fig. 10b, if the compliant joint along the upper face has a thickness of *s*, and *t* is the material thickness, the compliant joint along the core layer shall have a thickness (*s* + 4*t*). The one along the lower face shall have a thickness (*s* + 6*t*). The thickness of the transversal joints is *s*. According to the literature^29^, compliant joints of the desired span can be created that combine low bending and high tension/compression stiffness. Furthermore, under the typical boundary conditions, the larger compliant joint, located at the bottom of the sandwich panel, will mainly undergo tension stress in the working configuration and could be optimised accordingly. It is also assumed that the possible shear failure of the adhesive joining the two layers composing the facing of the sandwich panel will not be the dominant failure mode. Therefore, dealing with these aspects is postponed to a successive study, and the digital structural models presented in this paper assume rigid joints throughout.

The four equal columns, one of which is represented in Fig. 9b, are strict origami structures with a *C* -like cross-section and joined extra corner plates. Their joints may be either compliant joints or fasteners, according to the results from testing. In the following, the design of the proposed solution is presented, and the role of the design variables is discussed.

### Kinematic design

**Figure 11:**
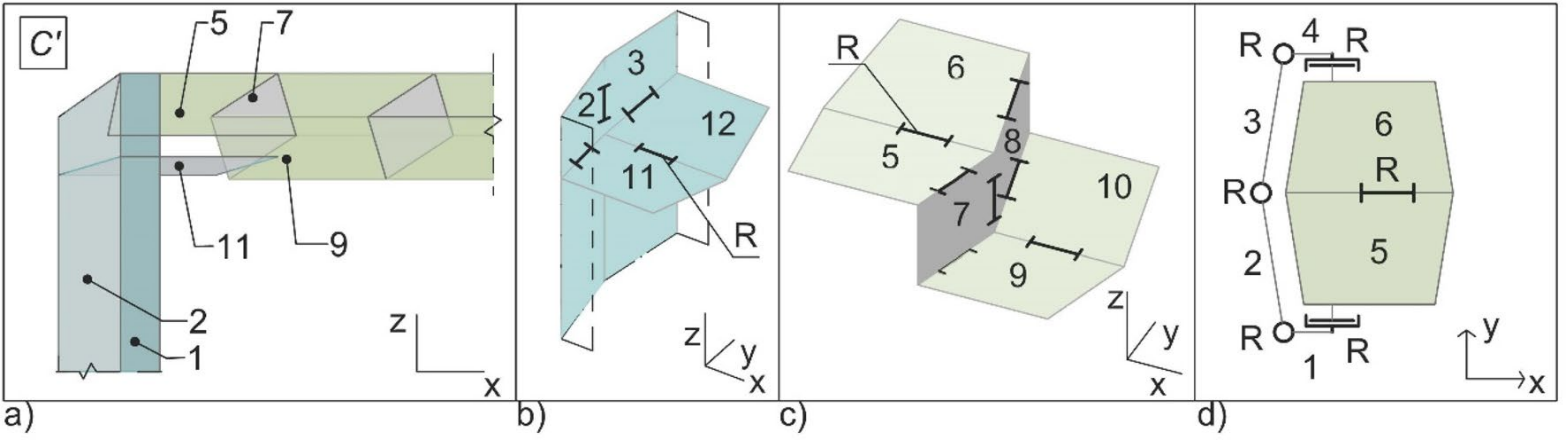
(**a**)The components of the mechanism at a generic folding stage *C*’; (**b**) and the kinematic models of the column; (**c**) a module of the floor; (**d**) and the column–floor connection.

The proposed origami-like modular system can be decomposed into three sub mechanisms: the sandwich floor, the connection between the floor and the columns, and the column (see Fig. 11). The floor is a modular assembly of Miura spherical linkages creating origami bellows^31^. The mechanism of one module is depicted in Fig. 11c and comprises two interconnected closed-loop linkages of six links connected by seven revolute joints (R). The concurrent joints’ directions create spherical mechanisms. Consequently, the degree-of-freedom λ of the space within which the mechanism operates is 3.

The mobility *M* of the mechanism can be calculated according to the classic Kutzbach formula:

1 $$M=\uplambda \cdot \left(N-1-j\right)+\sum \limits_{i=1}^{j}{f}_{i},$$

where *N* is the number of links, and *j* is the number of joints, each with freedom *f*_*i*_. This reads *M* = 3 (6 − 1 − 7) + 7 = 1. The additional modules composing the floors create further interconnected closed-loop linkages, and such do not change the mobility of the floor^19^. With reference to Fig. 9a, the value of the planar angle α must be α ≠ π/2; otherwise, the mechanism would reach an idle position during motion. In the proposed system, the angle is set constant throughout the plates. This ensures that the transversal horizontal edges of the floor stay horizontal during motion, which simplifies the connection with the columns. These are joined to the upper face of the floor. The kinematic model of the connection is represented in Fig. 11d. It is composed of six links, the revolute joints (R) connecting links labelled from 1 to 4, operating in the horizontal plane, and the ones connecting links labelled from 4 to 6, operating in the vertical plane. The degree-of-freedom λ of the space within which the mechanism operates is therefore 5. From Eq. (1), M = 5 (6 − 1 − 6) + 6 = 1. It can be noticed that the columns stick to the ground during motion.

**Figure 12:**
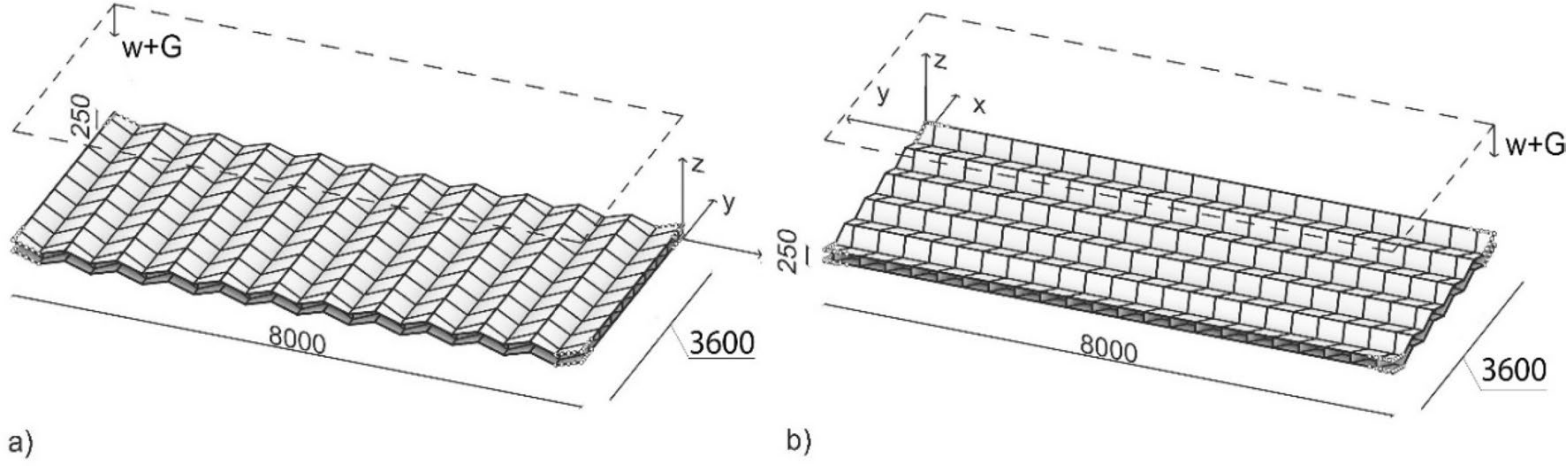
Sandwich floor with two-way corrugation. (**a**) Cells along transverse direction. (**b**) Cells along longitudinal direction.

The whole column further comprises additional corner plates, Fig. 12b, blue-coloured and slightly visible under the floors in Fig. 9. Not necessary for the foldability of the system, at the working configuration *C*, they are coplanar to the lower face of the foldable floor, to which they can be firmly connected with discrete joints. In such a way, they make it easy to block the mechanism and structurally strengthen the floor in the areas which will undergo the higher stress under the working loads. Meanwhile, at the columns, the lower face of the floor needs to be shorter than the upper one to avoid the floor and the column intersecting during folding. The extra corner plates form with the upper surface of the columns a spherical mechanism of the Miura type already employed in the floor, and Eq. (1) reads *M* = 3 (4 − 1 − 4) + 4 = 1. The whole mechanism is therefore a 1-DOF mechanism.

Looking to Fig. 9a, once set the linear dimensions *a, b,* and *c* of the plates, at any folding state *C’* the geometry of the floor is described by

2 $$\left\{\begin{array}{l}\Delta x=a\\ \Delta y{^{\prime}}= c\cdot sin(\tau {^{\prime}})\\ h{^{\prime}}= b+c\cdot cos\left(\tau {^{\prime}}\right)\cdot sin\left(\theta {^{\prime}}\right),\end{array}\right.$$

where θ′ = cos^−1^ (cos (α)/cos (τ′)). To ensure that the sandwich cell at the working configuration *C* has a rectangular profile, the dihedral angle τ must be τ = cos^−1^ (cos (α)/cos (π/4)). The motion is assumed to be in the domain 0 ≤ τ′ ≤ τ.

To ensure that the added corner plates are coplanar to the lower face of the floor at the target configuration *C*, it is imposed φ = τ at *C*, and consequently, the column has a linear dimension *e* = *c* ·sin(α). Furthermore, the height *b* of the core is

3 $$b\ge 0 \; \mathrm{ if }\; h \ge c \cdot cos(\tau ) \cdot sin(\pi /4).$$

### Structural analysis

**Figure 13:**
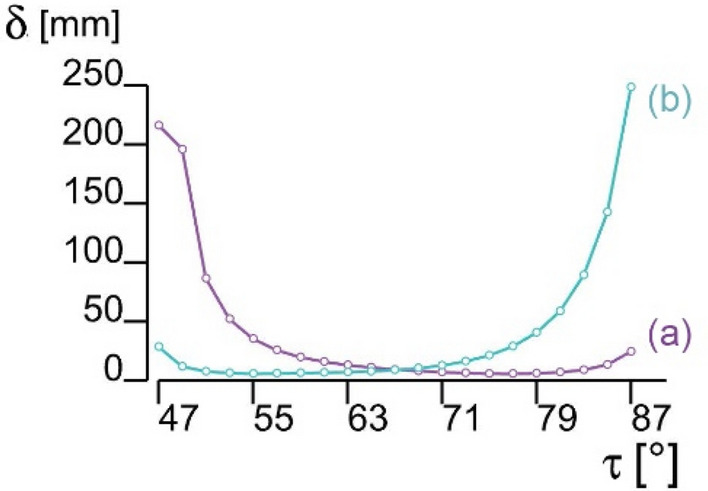
Deflection of the floor with varying angles [Case (a) and (b) are as in Fig. 12].

As by Eq. (2), the design variables are linked by a system of 3 equations in 4 unknowns. The role of the angle τ and such the angle α were analysed imposing a uniform distributed vertical load *w* = 1 kN/m^2^ in addition to self-weight G. In Fig. 12, structures with Δx = Δy = 40 mm, h = 20 mm, variable angles, and orientation of the trapezoidal cells respectively in longitudinal or transverse direction have been assumed. The resulting deflection is plotted in Fig. 13.

For low values of the height *b* of the core layer (for large values of the angles), the structure behaves like a simple concertina: if the rectangular cells are arranged transversely, Fig. 12a, it deforms excessively, whereas if the cells are oriented longitudinally, Fig. 12b, it produces a lower deflection d, Fig. 13. As the height *b* of the core increases, the value of the angle τ (and α) increases, and the structure is better oriented with the rectangular cells arranged transversally. This first solution reaches the minimum deflection among the two structures at an angle τ = 77° (α = 80.85°), which is adopted in the successive analyses. Overall, the orientation and angle of origami cells strongly influence the deflection magnitude of the proposed floor.

**Figure 14:**
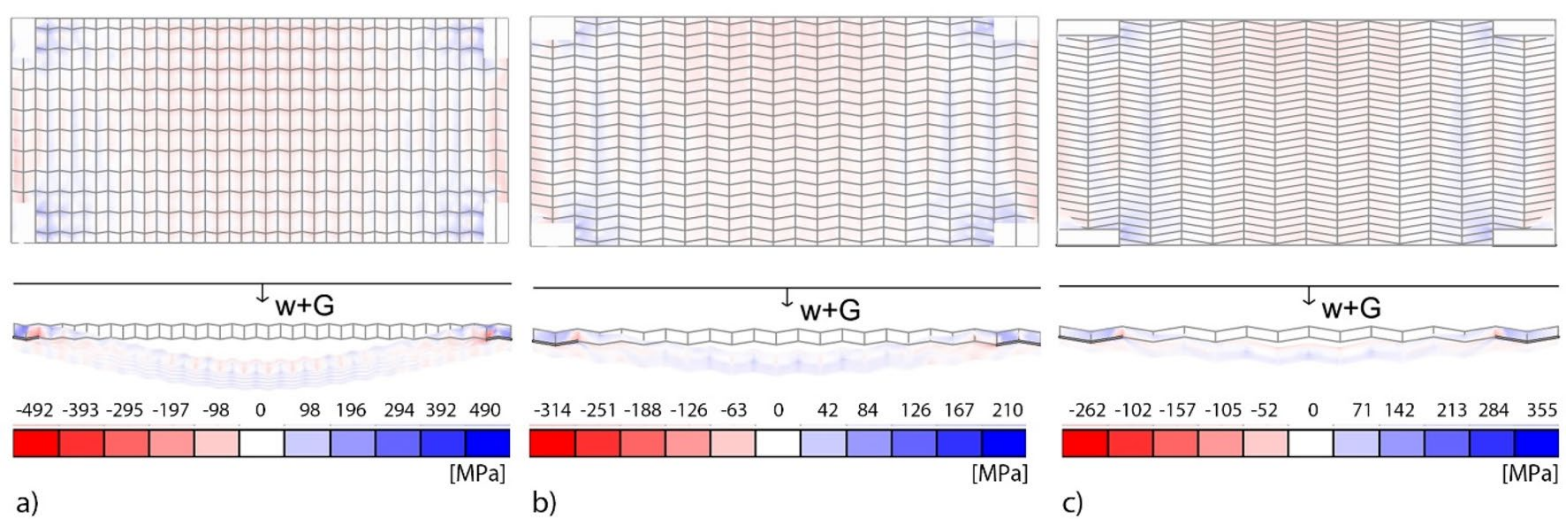
Von Mises stress distribution for Δy/Δx = 0.6 (**a**), Δy/Δx = 2.0 (**b**) and Δy/Δx = 4.0 (**c**). Blue represents tension and red compression.

**Figure 15:**
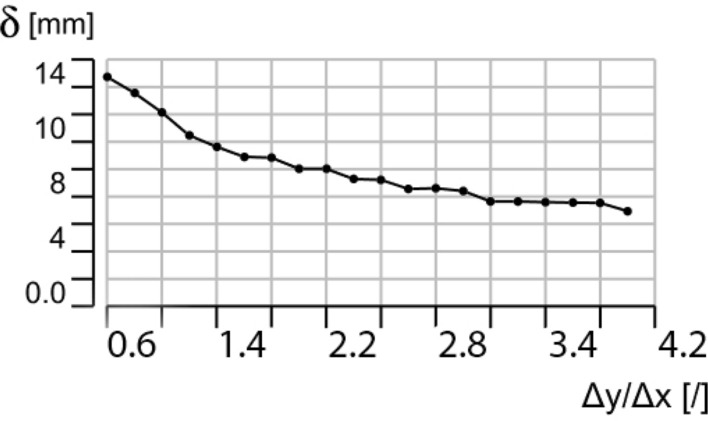
Maximum deflection d for increasing ratios Δy/Δx.

The second variable is the ratio Δ*y*/Δ*x*. As the ratio increases, the height of the core layer *b* decreases, and with it its risk of buckling. With reference to Fig. 14, a series of analyses were performed varying the ratio while assuming a constant value Δ*y*· Δ*x* = 62.5 10^3^ mm^2^. Since the distribution of stress in the cells depends on the boundary conditions, the rigid corner supports of the floor cover a rectangular surface of constant area *A*_*s*_ = 250· 10^3^ mm^2^ with the same ratio Δ*y*/Δ*x* between its sides. The structure is subjected to self-weight *G* and a uniformly distributed load *w* = 5 kN/m^2^. The distribution of stress for three reference configurations, respectively Δy/Δx equal to 0.6, 2, and 4 are depicted in Fig. 14, whereas the maximum deflection as a function of the ratio Δy/Δx is reported in Fig. 15.

In all cases, the core layer resulted to be the most stressed. The figures also show that, for low values of the ratio Δy/Δx, the maximum deflection d increases and so does the stress in the core layer, which yields, in favour of behaviour dominated by a beam action between the supports in the transversal direction. However, as the ratio increases, the stress increases in the face layers, which tend to plasticize near the supports.

**Figure 16:**
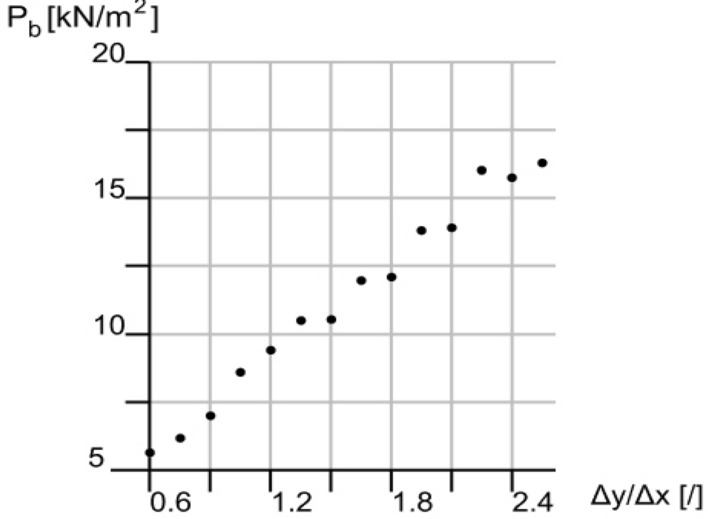
Variation of the buckling load for increasing ratios Δy/Δx.

To assess at first approximation the risk of buckling failure, the buckling load (see Fig. 16) has been calculated considering the geometric stiffness resulting from the second-order analysis. As one may expect, the first buckling mode in all analysed cases is a local buckling of the core layer near the supports. The buckling load has a progressive and remarkable increase with the increase of the ratio Δy/Δx, which suggests a high theoretical stiffness of the system when the cells have a proportion in the horizontal plane comparable to the horizontal proportions of the floor. However, the exact value of the buckling load shall be taken cautiously as the many possible manufacturing imperfections require further investigation.

In the complete solution, the rigid supports have been replaced with columns. As mentioned in discussing the mechanism, the columns have a *C-*shaped cross-section whose channel spans one concertina rib, that is a distance 2Δy. The relatively long channel that may result is stiffened by the fold line at its centre, which acts as a hinge during the unfolding process. The value of Δy determines the size of the cell, the last variable from Eq. (2).

A low value of Δy implies a high number of cells, a denser and more resistant core layer, and a slightly greater mass, whereas a high value increases the length of the channel of the *C*-shaped columns. Given the modularity of the system, it would be possible to locally decrease Δy to stiffen the structure in the areas of higher need, or to move the columns. At first approximation, given the symmetry of the panel and the usage of isotropic material, reverse deflection can similarly be treated, however, a detailed investigation addressing the asymmetry of the compliant joints would provide more reliable results.

## Conclusion

### Results

This paper has introduced the possibility of applying origami techniques to modular building systems. Modular building units are constructions composed of usually lightweight elements manufactured and preassembled off site. They ensure superior construction quality and a faster erection time compared to traditional structures. However, the transportability constraints limit their volume and the possible morphological variations. This paper has proposed to apply origami to overcome these issue. The deployability, constructability and structural performances of a corner -supported origami-like modular unit was presented. Given the lack of knowledge on origami for architectural engineering applications, and the novelty of the proposal, it was chosen to apply origami techniques to a corner supported modular building, without further variations. The proposed origami-like modular units perform well compared to the standard modular unit.

- The orientation and angle of the sandwich floor origami cells influence the deflection magnitude of the proposed floor.
- For low values of the ratio Δy/Δx, the maximum deflection increases and so does the stress in the core layer in favour of behaviour dominated by a beam action between the supports in the transversal direction.
- The buckling load calculated using second-order analysis for the origami sandwich floor increases with Δy/Δx ratio.
  An origami-like modular unit with Δy/Δx = 0.97, subjected to uniformly distributed gravity loads equal to 5 kN/m^2^ and self-weight, returned a maximum displacement equal to 66% the one of a standard MBA under the same boundary conditions, a lower Von Mises stress and had 90% of its mass.
- The proposed origami-like modular system allows the structure to fold into a very compact volume regardless of its longitudinal dimensions, leads to easy transportation, installation, and lightweight structure. The proposed system may be employed during post-disaster and emergencies (Covid-19).

### Future work and path to implementation

It is worth noting that the study at this stage is purely numerical and during the analysis the joints are assumed to be rigid. Further, the mechanics of compliant joints and the adhesive is not addressed for simplicity. However, this paper highlighted the lack of geometrical stiffness of the origami and possible ways to increase it at the point to be comparable with existing MBS structures. Overall, the principles discussed in the paper lead to further morphological investigations of novel modular origami-like units.

Future work of this research will include an exploration of kinematics and modularity to produce novel shapes, that can increase the desirability of the origami-like modular unit. To fully address the technology, it is recommended to test mechanically and via physical samples the energetics associated with the folding^32^. Further, the mechanics of the joints will be addressed through physical testing to calibrate the digital model.

## Data Availability

The datasets used and/or analysed during the current study available from the corresponding author on reasonable request.
